# The Mediating Effect of Body Mass Index on the Relationship between Cigarette Smoking and Atopic Sensitization in Chinese Adults

**DOI:** 10.3390/ijerph120303381

**Published:** 2015-03-23

**Authors:** Xiao Luo, Yupeng Wang, Zhiqiang Wang, Fuwen Cai, Biao Xie, Siyang Qu, Meina Liu

**Affiliations:** 1 Public Health College, Harbin Medical University, No.157, Baojian Road, Harbin City, Heilongjiang Province 150081, China; E-Mails: lxlx1218@163.com (X.L.); xiaoyu---007@163.com (Y.W.); caifuwen920@163.com (F.C.); xiexiexiebiaobiao@163.com (B.X.); qmiss1224@163.com (S.Q.); 2 School of Medicine, The University of Queensland, Health Sciences Building, Royal Brisbane & Women’s Hospital, Herston, QLD 4029, Australia; E-Mail: z.wang@uq.edu.au

**Keywords:** cigarette smoking, body mass index, mediator, atopy, adults

## Abstract

*Background:* It is unclear whether the relationship between cigarette smoking and atopy is mediated by body fat mass, such as the Body Mass Index (BMI). We assessed the mediating role of BMI on the relationship between smoking and atopy in Chinese adults. *Methods:* A hospital-based case-control study of 786 atopic cases and 2771 controls was conducted in adults aged 18 years or older from March 2010 to September 2014 in Harbin, China. Mediation models were used to estimate the indirect effects of smoking on atopic sensitization through BMI. *Results:* Compared to non-smokers, light smokers and moderate smokers had a lower risk of inhalant allergen sensitization. The indirect effect of smoking and sensitization to aeroallergens were only observed in light smokers (point estimate, −0.026; 95% CI, −0.062 to −0.004). The mediating roles of BMI on the relationships between smoking and other types of allergic sensitization were not statistically significant. *Conclusion:* BMI appeared to partially mediate the effect of light smoking on sensitization to aeroallergens. However, considering the other harmful health effects of cigarette smoking, the effective method to lower the incidence of atopy would be to decrease body fat mass by physical exercise and employing other more healthy ways of living rather than smoking.

## 1. Introduction

Atopic sensitization, as defined by positive specific Immunoglobulin E (IgE) test response to common food allergens and/or aeroallergens, is considered to be a vital stage in the pathogenesis of atopic disease [1]. It has been described that positive atopic responses in early life are significantly related to the development of atopic disorders in later life, such as hay fever and asthma [2]. Understanding the association of potential factors with atopic sensitization has important implications in developing strategies for the prevention of atopic disorders.

Cigarette smoking primarily suppresses T-helper lymphocyte type 2 (Th2) cytokine/chemokine responses in the lung [3], and decreases the incidence of atopic sensitization to aeroallergens [4,5,6,7,8,9,10,11]. Nevertheless, it is reasonable to assume that smoking affects the response to food allergens differently [12]. For instance, smoking is an important source of exposure to toxic elements such as lead (Pb), which is considered to be associated with increased odds of food sensitization in adults [13], but epidemiological findings are still inconsistent [14,15]. Meanwhile, adipose tissue, an important component of the human body, has been thought to influence the immune system and promote the development of atopic sensitization [16]. Body mass index (BMI), waist circumference (WC) and the waist-to-hip ratio (WHR) have been used as useful indicators of adiposity. Among them, BMI has been traditionally used as a surrogate marker of whole body adiposity and is the most frequently used diagnostic tool for obesity [17]. On the one hand, numerous studies report that current smokers are more likely to have lower mean BMI than non-smokers [18,19,20], and decreased fatness is associated with lower atopic sensitization risk [21,22]. On the other hand, although smokers have lower mean BMI, the risk of being obese seemed to increase with the daily number of cigarettes smoked [23,24,25], suggesting that the association between cigarette smoking and atopic sensitization might be mediated by BMI. Such a mediating role of BMI might differ depending on the daily consumption of cigarettes. Thus, it is necessary to clarify the role of BMI in the relationship between smoking categories and atopic sensitization to different types of allergens. 

Until now, no studies have examined the mediating role of BMI on the association of smoking status with atopic sensitization. Understanding this role is important for developing strategies for the prevention of allergic sensitization. If the effect of smoking on atopic sensitization was partially mediated by BMI, then an effective method to lower the incidence of atopy would be to decrease the body fat mass through physical exercise or other more healthy ways rather than starting to smoke. Therefore, the objective of our study was to examine the mediating effect of BMI on the association between smoking categories and atopic sensitization to different types of allergens in Chinese adults.

## 2. Material and Methods

### 2.1. Study Population

This is a hospital-based case-control study. A total of 3557 subjects aged 18 years or older at the Allergy Department of Affiliated Hospital of Harbin Medical University in Harbin, China, were enrolled in the study between March 2010 and September 2014. The participation rate was about 93.3%. Cases and controls were identified based on the results of specific IgE testing. Atopic sensitization cases were identified as those that tested positive to IgE for at least one of the common allergens. Controls were adults who visited the same department at the hospital for a health check-up during the aforementioned period and those who were negative in all specific IgE tests. All cases and controls were newly diagnosed of atopic sensitization and had no previous self-reported allergic disease history at enrollment. The study was approved by the Human Ethics Review Board; Harbin Medical University (Reference number is HMUIRB20120019). Written informed consents were obtained from all subjects involved in this study.

### 2.2. Information Collection

Demographic and lifestyle characteristics were recorded by trained interviewers. Information includes subject’s gender, age, educational level, self-reported family history of allergic disorders, alcohol consumption, smoking status, weight, height and specific IgE testing results. Trained interviewers conducted face-to-face interviews. Education level was categorized into three groups: illiterate or primary school, junior/senior secondary school and college, university. Self-reported family history of allergic disorder was present if the subject’s parents had any allergic diseases such as allergic rhinitis, allergic dermatitis, allergic asthma and food allergy. Regular alcohol drinking was defined as drinking more than twice per week for at least one year.

### 2.3. Definition of Smoking Categories

Definition of smoking status was based on World Health Organization criteria [26]. Those who had never smoked or had smoked for fewer than six months during their lifetime were classified as non-smokers. Former smokers were defined as those who had smoked for at least six months during their lifetime but had not smoked for at least 6 months before the face-to-face interview. Current smokers were defined as subjects who had smoked for at least six months during their lifetime and were smoking at the time of the interview. For current smokers, we also measured the average number of cigarettes smoked per day. Light, moderate and heavy smokers were defined as those smoking an average of 1–9 cigarettes, 10–20 cigarettes and at least 21 cigarettes per day, respectively.

### 2.4. Body Mass Index (BMI) Calculation

Height and weight were measured according to a standard protocol [27]. Individuals were required to dress in normal indoor clothing, without shoes, and then weighed to the nearest 0.1kg using a calibrated standard scale. Height was measured to the nearest 0.1cm using a stadiometer (Detector-Scales, Brooklyn, NY, USA). Body Mass Index (BMI) was calculated as weight (kg)/(height (m))^2^.

### 2.5. Specific IgE Test and Definition of Atopic Sensitization 

Serum samples were tested for allergen-specific IgE using the Allergy Screen system (Mediwiss Analytic GmbH, Germany). Twenty-two most common allergens in the northeast of China were assessed, including twelve aeroallergens and ten food allergens. Aeroallergens include *Dermatophagoides pteronyssinus*,cat and dog fur, common ragweed and mugwort, *Hop*, mould mixture (*Penicilliumnotatum*, *Cladosporium herbarum*, *Aspergillus fumigatus*, *Alternaria alternate*), tree pollen mixture (*Cottonwood*, *Elm*, *London Plane*, *Robur and Willow*), German cockroach, maize pollen, soybean pollen, oryza sativa pollen, wheat pollen and mulberry pollen. Food allergens included egg white/egg yolk, shrimp, crab, fish, milk, beef, pork, peanut, mango and pineapple. Atopic sensitization was considered positive when the concentration of at least one allergen-specific IgE was 0.35KU/L or greater. Atopic sensitization was then categorized into three groups based on the types of sensitized allergens: sensitized to any inhalant allergens only, sensitized to food allergens only and sensitized to both food and inhalant allergens. Those who were negative in all specific IgE tests were taken as controls.

### 2.6. Statistical Analysis

Descriptive statistical analysis was performed using SAS 9.2 statistical software (SAS Inc., Cary, NC, USA), and mediation analysis was conducted using SPSS 18.0 software (IBM, Corp, Armonk, NY, USA).Analysis of Variance (ANOVA) and chi-square tests were used to compare continuous and categorical variables, respectively, amongst different smoking categories. A two-sided *p* value of less than 0.05 was taken to indicate statistical significance.

**Figure 1:**
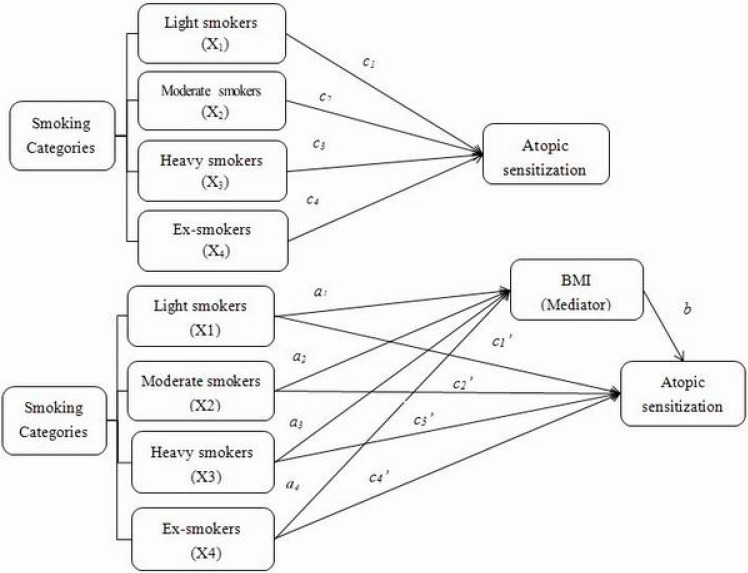
Model of the potential mediating effect of BMI on the association betweensmoking categories and atopic sensitization. The figure shows how to estimate the indirect effects through the potential mediator, of one smoking category (X_1_, X_2_, X_3_ and X_4_) on atopic sensitization compared to non-smoking category. Total effects (*c paths*) represent the overall association of a smoking category with atopic sensitization. Mediating effect occurred when the overall association could be partially (or fully) explained by the potential mediator (BMI). Association of the potential mediator with each smoking category (*a paths*) and the association with atopic sensitization independent of each smoking category were estimated. The products of *abs* (*a_1_b*, *a_2_b*, *a_3_b*, *a_4_b*) were used for estimating the indirect effects of BMI on the association between each smoking category and atopic sensitization. The *“c” paths’* represent the direct effects of each smoking category and atopic sensitization.

To understand whether the relationship between different smoking categories and atopic sensitization was mediated by BMI, mediation analysis was conducted [28], using the PROCESS Procedure for SPSS.As illustrated in Figure 1, the observed effect of the independent variables (different smoking categories) on the dependent variable (atopic sensitization) is called the total effect (*c* paths). The total effect includes two parts, a direct effect of the independent variable on the dependent variable (*c’* paths), and an indirect effect (*a* path + *b* path) of the independent variables on the dependent variable mediated by a third variable, known as the mediator. The indirect effects on atopic sensitization through the mediator (BMI) in one independent group compared to the reference group were estimated by the products of the coefficients of *a* paths and *b* paths, namely *ab*. 

We estimated: (1) *c* paths: compared to never smokers, the effect of being a light smoker (X_1_), moderate smoker (X_2_), heavy smoker (X_3_) or ex-smoker (X_4_) on atopic sensitization (Y) without including BMI (M) in the model;(2) *a* paths: the differences between each smoking category and reference group (never smoker) on the level of the potential mediator, such as BMI (M);and (3) *b* path: the association between mediator (BMI) and dependent variable (atopic sensitization) including independent variables (smoking categories) in the model. 

Coefficients for *a* paths were estimated using ordinary least square regressions and logistic regressions were used to estimate coefficients for *b* path, *c* paths and *c’* paths. Bootsrapping was conducted 10,000 times in order to obtain the bias-corrected 95% confidence interval (BC 95% CI) for the indirect effects, namely the product of *ab*s for each smoking category. Coefficients were considered statistically significant if the confidence intervals did not cross zero.

Potential confounders including sex, age, educational level, family allergic disease history and regular drinking were controlled when estimating the smoking status-atopy and the BMI-atopy relationships.

## 3. Results and Discussion

### 3.1. Result

#### 3.1.1. Characteristics of Study Subjects

A total of 3557 subjects (786 cases and 2771 controls) were included in the study. The786 cases were divided into the following three groups: (1) 587 cases who were sensitized to inhalant allergens only; (2) 82 to food allergens only; and (3) 117 to both food and inhalant allergens. Characteristics of the study subjects across the smoking categories are shown in Table 1. Compared to non-smokers, smokers were more likely to be males and older with lower educational level. Moreover, smokers were more likely to be regular drinkers than non-smokers. Mean BMI values were significantly different across smoking categories. Heavy and former smokers had higher BMI than non-smokers and light smokers. In addition, light and moderate smokers seemed to have a lower sensitization to aeroallergens than non-smokers. There were no significant differences in the family allergic disease history among smoking categories.

**Table 1:** Demographic and lifestyle characteristics of study subjects on the smoking status.

Characteristics	Non-Smokers	Current Smokers, Average Numbers of Cigarettes Per Day			Former Smokers	*p* Value ^a^
		Light Smokers (1–9)	Moderate Smokers (10–20)	Heavy Smokers (≥21)		
N	2942	184	249	53	129	
**Gender, %men**	16.76	55.43	72.29	84.91	75.97	<0.0001
**Age group %**						<0.0001
18–39	53.13	55.43	42.17	22.64	25.58	
40–59	41.13	38.59	53.01	67.92	54.26	
≥60	5.74	5.98	4.82	9.43	20.16	
**Educational level %**						<0.0001
1st	25.29	35.33	27.71	45.28	39.53	
2nd	38.38	32.61	52.21	33.96	34.11	
3rd	36.34	32.07	20.08	20.75	26.36	
**Family allergic disease history, %yes**	10.77	9.78	6.83	5.66	6.98	0.146
**Alcohol drinkers, %yes**	8.36	31.52	38.15	50.94	41.09	<0.0001
**Atopy(allergic to any allergens), %yes**	23.05	16.85	15.26	22.64	20.93	0.023
**Allergic toinhalant** **Allergens only %yes**	18.41	12.57	12.08	18.00	13.56	0.030
**Allergictofood** **allergens only %yes**	2.96	2.55	1.86	2.38	3.77	0.870
**Allergic to both food andaeroallergens %yes**	4.15	3.16	2.31	4.65	6.42	0.453
**BMI,kg/m^2^mean(SD)**	22.81 (3.59)	22.62 (3.50)	23.61 (4.66)	24.01 (3.32)	24.12 (3.56)	<0.0001

**^a^** Chi-square and ANOVA test were conducted for categorical variables and continuous variables, respectively. Abbreviations: Educational level. 1st, illiterate or primary school; 2nd, junior/senior secondary school; 3rd, college, university. SD, Standard Deviation.

**Table 2:** Association between each smoking category, atopic sensitization to different types of allergens and BMI in mediation model **^1^**.

Atopic Sensitization to Different Types of Allergens	BMI (Mediator)			Atopic Sensitization (Y )		
	Path	*β* (SE)	*p* Value	Path	*β* (SE)	*p* Value
**Allergic to inhalant** **Allergens only**						
Non-smokers		Reference			Reference	
Light smokers(X_1_)	a_1_	−0.906 (0.279)	0.001	c_1_	−0.476 (0.239)	0.046
Moderate smokers(X_2_)	a_2_	−0.338 (0.252)	0.180	c_2_	−0.529 (0.216)	0.014
Heavy smokers(X_3_)	a_3_	−0.397 (0.512)	0.438	c_3_	−0.033 (0.383)	0.932
Former smokers (X_4_)	a_4_	−0.156 (0.345)	0.651	c_4_	−0.363 (0.286)	0.204
BMI, kg/m^2^ (M)	b	----	----	----	0.029 (0.013)	0.022
**Allergic to both food andinhalant allergens**						
Non-smokers		Reference			Reference	
Light smokers(X_1_)	a_1_	−0.944 (0.289)	0.001	c_1_	−0.729 (0.479)	0.128
Moderate smokers(X_2_)	a_2_	−0.284 (0.261)	0.278	c_2_	−1.165 (0.482)	0.016
Heavy smokers(X_3_)	a_3_	0.030 (0.541)	0.056	c_3_	−0.528 (0.753)	0.483
Former smokers (X_4_)	a_4_	−0.251 (0.354)	0.478	c_4_	−0.095 (0.434)	0.828
BMI, kg/m^2^(M)	b	----	----	----	0.013 (0.027)	0.629

**^1^** Mediation analysis adjusted for covariates, including sex, age, educational level, familyallergic disease history and alcohol consumption. Abbreviations: SE, Standard Error; BMI: Body Mass Index.

#### 3.1.2. Associations between Each Smoking Category, BMI and Atopic Sensitization to Different Types of Allergens 

Table 2 presents the associations among smoking status, BMI and atopic sensitization. Light and moderate smokers were both associated with a lower risk of atopic sensitization to aeroallergens only, compared with non-smokers, after adjusting for other confounders (*β* (SE) = −0.476 (0.239) and −0.529 (0.216), *p =* 0.046 and 0.014, for light and moderate smoking, respectively). Meanwhile, a significant inverse association between smoking categories and BMI levels was found in light smokers (*β* (SE) = −0.906 (0.279), *p =* 0.001). There was a positive association between BMI and atopic sensitization to aeroallergens only (*β* (SE) = 0.029 (0.013), *p =* 0.022), which was consistent with a previous study [29]. Consequently, an analysis was carried out to determine whether smoking categories had different impact on the responses to other types of allergens. We only found an inverse association between moderate smoking and sensitization to both food and inhalant allergens (*β* (SE) = −1.165 (0.482), *p =* 0.016). There was no relationship between any smoking categories and sensitization to food allergens only (Supplement Table S1).

#### 3.1.3. Indirect and Direct Effect, through Potential Mediator, of Smoking Categories on Atopic Sensitizationto Different Types of Allergens

The indirect effects of smoking categories on atopic sensitization to different types of allergen are shown in Table 3. We found that BMI only mediated the association between light smoking and atopic sensitization to aeroallergens only, and the indirect effect of a light smoker on atopic sensitization was statistically significant (point estimate (SE): −0.026 (0.015), BC 95% CI: −0.062 to −0.004). In contrast, no indirect effects of smoking categories on sensitization to both food and aeroallergens were found. In addition, we also estimated the mediation role of BMI on the association between each smoking category on sensitization to food allergens only. However, no indirect effect was observed (Supplement Table S2).

**Table 3:** Indirect and direct effect, through potential mediator, of smoking categories on atopic sensitization **^1^**.

Atopic Sensitization to Different Types of Allergens	Indirect Effect of Smoking on Atopic Sensitization to Different Types of Allergens *(ab paths)*			Direct Effect of Smoking on Atopic Sensitization to Different Types of Allergens *(c’ paths)*		
	Path	Point Estimate (SE)	BC 95% CI	Path	Point Estimate (SE)	BC 95% CI
**Only allergic to aeroallergens**						
Non-smokers		Reference			Reference	
Light smokers (X_1_)	a_1_b	−0.026 (0.015)	−0.062 to −0.004	c’_1_	−0.452 (0.239)	−0.921 to 0.018
Moderate smokers (X_2_)	a_2_b	−0.010 (0.013)	−0.043 to 0.008	c’_2_	−0.525 (0.217)	−0.950 to −0.101
Heavy smokers (X_3_)	a_3_b	−0.011 (0.017)	−0.059 to 0.014	c’_3_	−0.021 (0.384)	−0.773 to 0.731
Former smokers (X_4_)	a_4_b	−0.005 (0.011)	−0.034 to 0.013	c’_4_	−0.361 (0.286)	−0.921 to 0.200
**Allergic to both food and aeroallergens**						
Non-smokers		Reference			Reference	
Light smokers (X_1_)	a_1_b	−0.012 (0.029)	−0.071 to 0.043	c’_1_	−0.716 (0.480)	−1.657 to 0.227
Moderate smokers (X_2_)	a_2_b	−0.004 (0.015)	−0.051 to 0.015	c’_2_	−1.160 (0.482)	−2.105 to −0.215
Heavy smokers (X_3_)	a_3_b	0.0004 (0.017)	−0.031 to 0.040	c’_3_	−0.524 (0.753)	−1.999 to 0.951
Former smokers (X_4_)	a_4_b	−0.003 (0.014)	−0.048 to 0.014	c’_4_	−0.092 (0.434)	−0.942 to 0.758

**^1^** Mediation analysis adjusted for covariates, including sex, age, educational level, family allergic disease history and alcohol consumption. Abbreviations: SE, Standard Error; BMI: Body Mass Index. BC 95% CI, Bias-Corrected 95% Confidence Interval.

### 3.2. Discussion

In this study, we found that light smokers and moderate smokers had lower risk of having allergic sensitization to aeroallergens compared with non-smokers. More importantly, we demonstrated the mediating effect of BMI on the association between light smoking and aeroallergen sensitization. To the best of our knowledge, this is the first study that examined the mediating role of BMI on the relationship between cigarette smoking and atopic sensitization. 

Our findings indicate that current light and moderate smokers have a lower risk of experiencing aeroallergen sensitization than do never smokers. Previous studies examining the relationship between cigarette smoking and atopic sensitization reported that smoking was associated with a lower risk of atopic sensitization to aeroallergens. However, no studies have substantiated the relationship between average number of cigarettes smoked and atopic sensitization, even though some of these had recorded information about the amount of cigarettes consumed [4,8,9]. The amount of cigarettes consumed might affect the immune system and its functioning, which might lead to different responses to allergens. 

In our study, we demonstrated that light and moderate smoking is related to a decreased risk of atopy in adults. However, for heavy and former smokers, such an association was not observed. The main reason for this non-significant association might be due to the small number of these two smoking categories in this study. Efforts should be made to increase the number of heavy and former smokers in future studies to assess this relationship

Smoking might affect the response to food allergens differently than it affects the response to aeroallergens [12], and this association might differ with differing daily consumptions of cigarettes. In our study, no relationship between smoking categories and sensitization to food allergens only was observed. This finding should be interpreted with caution. It is worth noting that the sample size of food sensitization cases is relatively smaller than those of other types of sensitization. Moreover, smoking might decrease the risk of exposure to food allergens by reducing food intake, which could contribute to the observed negative relationship between cigarette smoking and sensitization to food only. Although we found that moderate smoking was associated with lower risk of sensitization to food and aeroallergens, the precise mechanism underlying this relationship is still unknown. Further studies are not only required to increase the sample size, but also to focus on the precise mechanisms underlying the association between smoking categories, sensitization to food allergens, and sensitization to both food and inhalant allergens. 

This study revealed, for the first time, that BMI might play a mediating role in the association between light smoking and sensitization to aeroallergens. This evidence suggests that cigarette smoking could influence atopic sensitization through an indirect pathway. Several studies have demonstrated that light smokers are likely to have lower BMI than non-smokers due to the increased metabolic rate [30,31,32] or restricted caloric absorption [33] precipitated by smoking. Epidemiological studies have shown that, compared with obese subjects, subjects within a normal BMI range have lower risk of atopic sensitization [21,22,34]. Therefore, the decreased atopic sensitization in light smokers might be partially mediated by BMI. For moderate and heavy smokers, the risk of being obese increased with the numbers of cigarettes smoked per day. Adipose tissue produces high serum concentrations of various pro-inflammatory cytokines, chemokines and adipokines [35], contributing to the skewing of the immune system towards a Th2 cytokine profile and thereby leading to an increased risk of atopic sensitization [36]. This may be the reason that the mediating role of BMI on the relationship between moderate smoking or heavy smoking and atopic sensitization differs from that for the relationship between light smoking and atopic sensitization. However, we did not find any direct evidence in this study.

Unlike previous studies in which BMI was treated as a categorical variable with three states (normal, overweight, and obese), we treated it as a continuous variable when exploring its mediating role on the relationship between smoking and atopic sensitization. This enabled us to examine the changes to the risk of atopic sensitization corresponding to per 1kg/m^2^ increments in BMI levels, especially for current smokers. Interestingly, we found that among studies that explored the relationship between cigarette smoking and atopic sensitization, only one considered the role of body fat mass when fitting the cigarette smoking-atopic sensitization model [6]. In that study, BMI was taken as a covariate when exploring the association between smoking status and specific IgE positivity for different age categories. Therefore, there is need to consider the role of BMI on the association between cigarette smoking and atopic sensitization when fitting the smoking-atopy model.

The prevalence of smoking in our population is different from that of the Global Adult Tobacco Survey (GATS) in the Chinese population in 2010 [37].This difference could be due to different definitions of smoking, given that the two studies had different original objectives, even though definitions of smoking habits in both studies were based on the same World Health Organization criteria. Further studies are required to assess the generalizability of our findings using nationally representative samples.

The strengths of our study include a relatively large sample size for male and female cases and controls; and it is the first study to clarify the mediating effect of BMI on the association between cigarette smoking and atopic sensitization in Chinese adults. The primary limitations the cross-sectional design, which does not allow us to establish temporality between the factors of interest and atopic sensitization. We assumed that smoking leads to lower BMI, which then contributes to lowering the incidence of atopic sensitization. It is still possible that obese or overweight subjects might smoke for the purpose of losing weight, especially in women [38,39]. However, this is unlikely in our study because most of our smokers are males. There is no evidence that Chinese men pickup smoking for the purpose of losing weight. Data obtained from prospective studies may be useful to further verify our findings. Secondly, we did not measure other body adiposity indicators, such as waist circumference (WC), and waist-to-hip ratio (WHR). Those measurements are considered to be better correlated with tobacco usage than BMI [40]; and might have different roles in the association between cigarette smoking and atopic sensitization. Thirdly, we did not obtain information about other risk factors for atopic sensitization, such as dietary pattern, which might be different between smokers and non-smokers, and contribute to the association between body weight and atopic sensitization. Finally, the relationships are probably related to more than a single mediator variable. Future studies examining other potential mediators will provide new viewpoints for the association between cigarette smoking and atopic sensitization.

## 4. Conclusions

In summary, in addition to confirming the relationship between cigarette smoking and atopic sensitization, our study, for the first time, has demonstrated that BMI might mediate the association between light smoking and sensitization to aeroallergens only, in Chinese adults. However, considering the demonstrated harmful effects of cigarettes, a more appropriate method to lower the incidence of atopic sensitization would be to control body weight by engaging in physical exercise and other healthy lifestyle choices, rather than by smoking. 

## Supplementary Files

Supplementary File 1
