# Supplementary material for: The Mediating Effect of Body Mass Index on the Relationship between Cigarette Smoking and Atopic Sensitization in Chinese Adults
Source: Int J Environ Res Public Health. 2015 Mar 23;12(3):3381–94. doi: 10.3390/ijerph120303381 (PMC4377972; doi:10.3390/ijerph120303381)
Supplement: Supplementary File 1 [file ijerph-12-03381-s001.pdf]

The Mediating Effect of Body Mass Index on the Relationship between Cigarette Smoking and Atopic Sensitization in Chinese Adults

Table S1. Association between each smoking category, different types of atopic sensitization and BMI in mediation model [1].

Atopic Sensitization to Different Types of Allergens	BMI (Mediator)			Atopic Sensitization (Y)		
	**Path**	***β*** **(SE)**	***p*** **Value**	**Path**	***β*** **(SE)**	***p*** **Value**
**Allergic to food allergens only**						
Non-smokers		Reference			Reference	
Light smokers(X_1)	a_1	−0.890 (0.287)	0.002	c_1	−0.130 (0.534)	0.809
Moderate smokers(X_2)	a_2	−0.261 (0.261)	0.319	c_2	−0.444 (0.549)	0.419
Heavy smokers(X_3)	a_3	0.055 (0.543)	0.919	c_3	−0.106 (1.043)	0.919
Former smokers (X_4)	a_4	−0.309 (0.356)	0.386	c_4	0.396 (0.561)	0.481
BMI, kg/m^2 (M)	b	--------	--------	--------	0.062(0.027)	0.025

[1] Mediation analysis adjusted for covariates, including sex, age, educational level, family allergic disease history and alcohol consumption. Abbreviations: SE, Standard Error; BMI: Body Mass Index.

Table S2. Indirect and direct effect, through potential mediator, of smoking categories on atopic sensitization [1].

Atopic Sensitization to Different Types of Allergens	Indirect Effect of Smoking on Atopic Sensitization (*ab* Paths)			Direct Effect of Smoking on Atopic Sensitization (*c'* Paths)		
	**Path**	**Point Estimate (SE)**	**BC 95% CI**	**Path**	**Point Estimate (SE)**	**BC 95% CI**
**Allergic to food allergens only**						
Non-smokers		Reference			Reference	
Light smokers (X_1)	a_1b	−0.055 (0.030)	−0.139 to −0.006	c'_1	−0.083 (0.535)	−1.132 to 0.967
Moderate smokers (X_2)	a_2b	−0.016 (0.029)	−0.092 to 0.028	c'_2	−0.480 (0.551)	−1.561 to 0.600
Heavy smokers (X_3)	a_3b	0.003 (0.038)	−0.072 to 0.086	c'_3	−0.118 (1.042)	−2.161 to 1.924
Former smokers (X_4)	a_4b	−0.019 (0.028)	−0.098 to 0.022	c'_4	0.396 (0.562)	−0.705 to 1.496

[1] Mediation analysis adjusted for covariates, including sex, age, educational level, family allergic disease history and alcohol consumption. Abbreviations: SE, Standard Error; BMI: Body Mass Index. BC 95% CI, Bias-Corrected 95% Confidence Interval.

Table S3. Characteristics of atopic sensitization cases and hospital health controls in Chinese adults.

	**Case ***	**Control**	***p* Value [a]**
N	786	2771	
Gender % men	27.23	25.41	0.303
Age group %			0.174
18–39	53.69	50.27	
40–59	40.97	43.20	
≥60	5.34	6.53	
Educational level %			0.012
1st	27.23	26.67	
2nd	34.61	40.02	
3rd	38.17	33.41	
Family allergic disease history % yes	10.05	10.29	0.848
Alcohol drinkers % yes	12.85	13.64	0.566
Smoking categories			0.023
Non-smokers	86.26	81.70	
Light smokers [1]	3.94	5.52	
Moderate Smoker [2]	4.83	7.61	
Heavy smokers [3]	1.53	1.48	
Former smokers	3.44	3.68	
BMI, kg/m^2 mean (SD)	23.20(4.11)	22.84(3.54)	0.025

* Cases were identified as those that tested positive to IgE for at least one of the common allergens; [a] Chi-square and *t* test were conducted for categorical variables and continuous variables, respectively; [1] Light smokers: smoking an average of 1–9 cigarettes per day; [2] Moderate smokers: smoking an average of 10–20 cigarettes per day; [3] Heavy smokers: smoking an average of 21 and more cigarettes per day. Abbreviations: Educational level. 1st, illiterate or primary school; 2nd, junior/senior secondary school; 3rd, college, university. SD, Standard Deviation.

© 2015 by the authors; licensee MDPI, Basel, Switzerland. This article is an open access article distributed under the terms and conditions of the Creative Commons Attribution license (http://creativecommons.org/licenses/by/4.0/).
